# Metastatic Testicular Mixed Germ Cell Tumor Presenting as Posterior Scrotal Pain in the Emergency Department: A Case Report

**DOI:** 10.7759/cureus.46072

**Published:** 2023-09-27

**Authors:** Austin Corpuz, Kindalem Fentie, Dylan Thomas, Misbha Anjum, Joseph Bear

**Affiliations:** 1 Medicine, Trinity School of Medicine, Warner Robins, USA; 2 Surgery, Piedmont Macon Medical Center, Macon, USA

**Keywords:** scrotal pain, testicular cancer metastasis, testicular germ cell tumors, testicular mass, mixed germ cell tumor

## Abstract

Testicular neoplasms, or testicular cancer, are not typically seen in the emergency department (ED) since their presentation involves a painless hard mass that emerges slowly over time. Uncommon presentation of testicular neoplasm to the ED with acute onset of scrotal pain may present challenges as an incomplete physical examination without supplemental imaging and laboratory workup may overlook the diagnosis of testicular neoplasm. As a result, a delay in proper treatment may occur. Early recognition of testicular neoplasm can decrease morbidity and mortality and improve overall patient survival. Here, we present a case of a 32-year-old male who presented in the ED with an acute onset of testicular pain localized on the posterior right side of the scrotum. Despite the unusual presentation, a complete physical examination, including a complete genitourinary system exam, was performed. During the physical examination, a high index of suspicion for testicular neoplasm was present. Necessary imaging and laboratory workup were ordered. Based on the findings, testicular neoplasm was highly suspected. Thus, surgical intervention was pursued to remove the suspicious mass and pathology revealed a mixed germ cell tumor. Further imaging and laboratory workup showed metastasis into other organ systems, and medical management was chosen to treat the metastatic neoplasm systemically.

## Introduction

Testicular tumors are relatively rare, accounting for 1-2% of cancer among men in the United States (US), with a persistent increase in incidence over the past 10 years and doubled over the past 40 years in most countries worldwide. However, the mortality rate has declined across the board owing to advancements in chemotherapy. Testicular tumor is the most common solid malignant tumor in young men aged 15-35 years in the US [1,2].

Testicular tumors are classified into two groups according to their pathology: (1) germ cell tumors (GCTs) and (2) non-GCTs. GCTs originate from the germinal epithelium. Non-GCTs originate from the gonadal stroma. Tumors originating from germ cells comprise most of the testicular tumors, approximately 95% of them. GCTs are further categorized into two categories: (1) seminomatous, which comprise about 45%, and (2) non-seminomatous tumors, which may be prevalent among the population either in their pure or combined form [3].

Mixed GCT is a type of testicular tumor with two or more GCT types within a single mass. Mixed GCTs account for one-third of all testicular GCTs [4]. Testicular tumors typically present with unilateral painless testicular nodules or swelling. A dull lower abdominal pain could sometimes be presenting symptoms of a testicular tumor, whereas testicular torsion, varicocele, or epididymitis typically presents with acute testicular pain.

The etiology of testicular tumors is multifactorial, including genetic and environmental risk factors. Based on a case-control study in the Czech Republic, a multivariate analysis of risk factors for testicular cancer in hospitals showed multivariate-adjusted OR for all testicular germ cell cancer: atrophic testis (5.9), family history of prostate cancer (4.8), cryptorchidism (3.8), and in univariate analyses (OR) cryptorchidism with delayed orchidopexy after five years old (5.7) [5].

The U.S. Preventive Services Task Force, National Cancer Institute, and American Academy of Family Physicians recommend against routine screening for testicular cancer in asymptomatic adolescents and adults due to its low prevalence, limited accuracy of screening tests, and no evidence of incremental benefit of screening.

Testicular cancer is curable even when metastasized. The overall five-year survival rate is 97% with effective treatment [6].

## Case presentation

A 32-year-old male presented to the ED for an acute onset of a painful swelling localized on the posterior side of the right scrotum. The patient was sexually active, a former one-half pack per day smoker of five years, and reported being hit with a cardboard box on the scrotum a few weeks ago. The patient described an intermittent history of unilateral swelling and painful sensations emanating from the right posterior scrotum with accompanying dysuria. He admitted to recent changes in urinary frequency with normal urine flow upon voiding but denied any history of hematuria. Furthermore, the patient denied a recent onset of fever, weight loss, and general fatigue. An initial impression of acute epididymitis was made. Upon performing a physical examination, a unilateral, warm, erythematous, firm, right testicular mass was discovered in a normal orientation.

**Figure 1:**
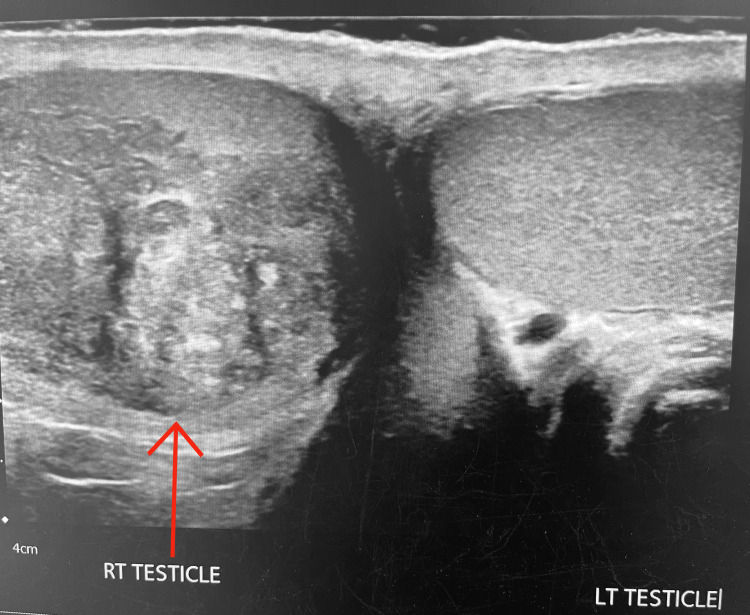
Heterogeneous intratesticular mass with a small right hydrocele was present on the right testicle measuring 3.1 x 3.4 x 2.6 cm.

An ultrasound was performed, which revealed a 3.1 x 3.4 x 2.6 cm heterogeneous intratesticular mass with a small hydrocele, which was present on the right testicle (Figure 1). The ultrasound showed the presence of arterial blood flow on the right testicle without evidence of anatomical testicular displacement. A diagnosis of a testicular neoplasm was then highly suspected, and surgery was pursued.

Prior to surgery, an initial blood workup of AFP, HCG, and LDH revealed an elevation in AFP.

A right radical inguinal orchiectomy was performed and was sent for histology review. Pathology revealed a 2.8 x 3.7 x 4.0 cm testicular mass composed of an advanced mixed GCT with seminomatous component (15%), embryonal carcinoma (50%), yolk sac tumor (15%), and malignant teratoma (20%) limited to the testes with good risk. There were negative margins showing no lymphovascular invasion. The pathologic stage classification is pT1b, cN1 M1a, and S1.

**Figure 2:**
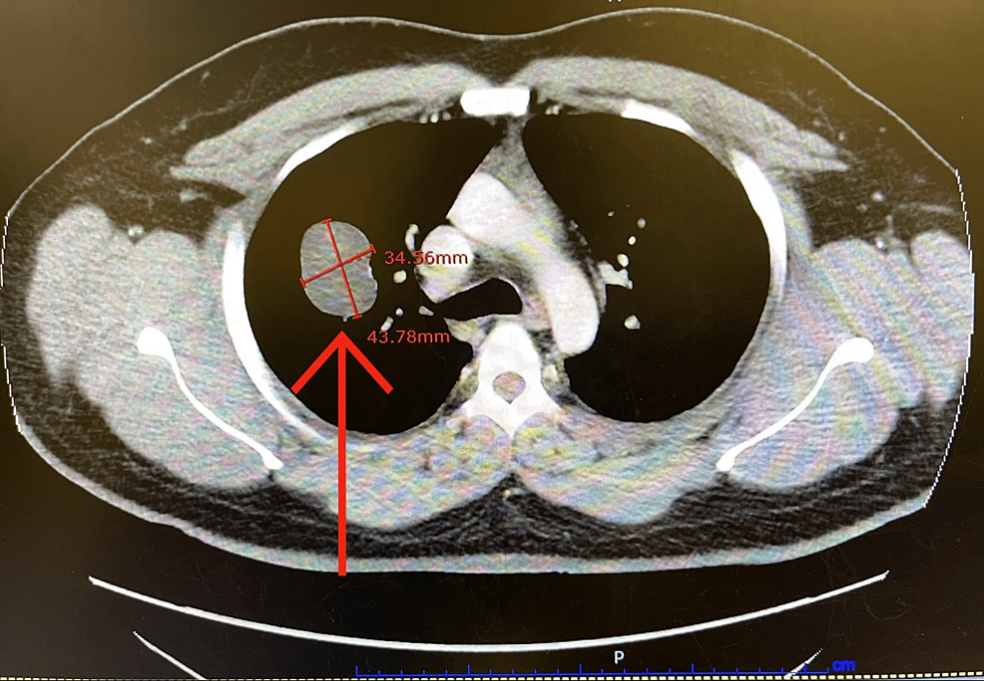
A large indeterminate lobular, right upper lobe lung cancer, measuring 4.4 cm.

Given the pathological report, post-orchiectomy staging/metastatic workup with CT chest, abdomen, and pelvis scan with and without contrast revealed a large indeterminate lobular, right upper lobe lung cancer, measuring 4.4 cm (Figure 2). There was also infiltration of the right lower abdominal wall, with thickening of the adjacent skin. There were borderline enlarged right external iliac chain lymph nodes.

The patient had a PET scan, which reported a right upper lobe mass, measuring 3.33 cm x 3.76 cm with a maximum SUV of 7.3, which was consistent with metastatic disease. Additionally, there was a metastatic lesion in the right hemipelvis measuring 1.6 x 1.26 cm with an SUV of 5.1.

Blood workup was rechecked, which showed that AFP was elevated at 350.9 U/L, LDH was 111 U/L, and beta-hCG was within normal limits. The patient was referred and seen by the medical oncology team for further management. The patient was started on first-line chemotherapy with bleomycin, etoposide, and cisplatin and scheduled for a PET scan upon completion of treatment.

## Discussion

Acute testicular pain is a common presentation in young adults, primarily due to epididymitis and testicular torsion, but rarely due to testicular tumors. In comparison to the common incidence of torsion per year, testicular GCTs are relatively rare, accounting for only 1-2% of cancers among men in the US [7].

The common presentation of testicular tumor is a painless testicular nodule or swelling with a negative transillumination test, unlike our patient who initially presented with testicular pain, which usually leads to diagnostic delay due to uncommon presentation. Diagnostic delay is a very well-recognized phenomenon in the literature with previous studies reporting that up to one-third of testicular tumors were initially misdiagnosed as epididymitis or hydrocele [8]. An example of a case in which a tumor presented with acute pain leading to a misdiagnosis was reported by Alrabeeah et al. in 2017 who described a case of testicular seminoma presenting as an acute scrotum pain [9]. They described the causes that led to the masking of the diagnosis, in that case, which were the inability to properly assess the testis on physical examination caused by the surrounding scrotal edema and the nonspecific changes on ultrasound imaging, which led to a provisional diagnosis of epididymo-orchitis. This ultimately led to a delay in the ideal management.

There are case reports that reported testicular tumors that presented with acute testicular pain; in most of them, the pain is attributed to the tumor causing a torsion due to the mass effect or intratesticular bleeding. Another example of a case in which a testis tumor in an adult, presenting with torsion of the testis, was reported by Seaman et al. [10] This article suggests that testicular neoplasm should not be overlooked as a differential diagnosis when suspect testicular torsion on a patient presents with sudden onset of testicular pain.

In our case, an ultrasound done for testicular pain revealed a 3.1 x 3.4 x 2.6 cm heterogeneous right intratesticular mass and elevated tumor markers for which our patient underwent radical right orchiectomy with a lateral inguinal approach; the finding is pT1b mixed GCT with seminomatous component (15%), embryonal carcinoma (50%), yolk sac tumor (15%), and malignant teratoma (20%) limited to the testes.

A retrospective case note analysis of patients undergoing radical orchiectomy over an 11-year year period in Hull, UK, shows that presentation with pain did not appear to correlate with any particular histological subtype of neoplasm or stage of disease. However, those presenting with GCTs and testicular pain were more likely to suffer disease relapse than those presenting with painless testicular enlargement (1.6% compared to 2.6%) [11], although, in our patient, it was his first diagnosis. His initial presentation of pain does put him at higher risk for relapse.

## Conclusions

Scrotal pain is an uncommon presentation of testicular tumors, as presented in our case report. Our recommendation upon reviewing our case would be testicular neoplasm should not be overlooked for a patient presenting to the ED with a sudden onset of pain localized on the posterior scrotum as this could change the management and outcomes. In addition to a complete genitourinary physical exam, physicians should include testicular tumors in their differential diagnosis and obtain imaging with tumor markers, such as AFP, LDH, and beta-HCG earlier in the workup. These steps may help identify testicular tumors early, which could result in better treatment outcomes.
